# The downregulation of PRDM1/Blimp-1 is associated with aberrant expression of miR-223 in extranodal NK/T-cell lymphoma, nasal type

**DOI:** 10.1186/1756-9966-33-7

**Published:** 2014-01-17

**Authors:** Li Liang, Lin Nong, Shuang Zhang, Jing Zhao, Hongjuan Ti, Ying Dong, Bo Zhang, Ting Li

**Affiliations:** 1 Department of Pathology, Peking University First Hospital, Beijing 100034, China; 2 Department of Pathology, Haidian Hospital of Beijing, Beijing 100080, China; 3 Department of Pathology, Peking University Health Science Center, Beijing 100191, China

**Keywords:** Extranodal NK/T-cell lymphoma, Nasal type, PRDM1, miR-223

## Abstract

**Background:**

The mechanism for inactivation of positive regulatory domain containing I (PRDM1), a newly identified tumour suppressor gene in extranodal NK/T-cell lymphoma, nasal type (EN-NK/T-NT) has not been well defined. The aim of the present study was to investigate the expression of PRDM1 in EN-NK/T-NT and analyse its downregulation by miRNAs.

**Methods:**

PRDM1 and miRNA expression were evaluated in EN-NK/T-NT samples by immunohistochemical analysis, qRT-PCR, and in situ hybridisation. Luciferase assays were performed to verify the direct binding of miR-223 to the 3′-untranslated region of PRDM1 mRNA. In addition, the effect of miR-223 on PRDM1 expression was assessed in NK/T lymphoma cell lines by transfecting a miR-223 mimic or inhibitor to increase or decrease the effective expression of miR-223. Overall survival and failure-free survival in EN-NK/T-NT patients were analysed using Kaplan-Meier single-factor analysis and the log-rank test.

**Results:**

Investigation of the downregulation of PRDM1 in EN-NK/T-NT cases revealed that PRDM1-positive staining might be a favourable predictor of overall survival and failure-free survival in EN-NK/T-NT patients. However, the negative staining of PRDM1 usually presented transcripts, suggesting a possible post-transcriptional regulation. miR-223 and its putative target gene, PRDM1, exhibited opposite patterns of expression in EN-NK/T-NT tissues and cell lines. Moreover, PRDM1 was identified as a direct target gene of miR-223 by luciferase assays. The ectopic expression of miR-223 led to the downregulation of the PRDM1 protein in the NK/T-cell lymphoma cell line, whereas a decrease in miR-223 restored the level of PRDM1 protein.

**Conclusions:**

Our findings reveal that the downregulation of the tumour suppressor PRDM1 in EN-NK/T-NT samples is mediated by miR-223 and that PRDM1-positive staining might have prognostic value for evaluating the clinical outcome of EN-NK/T-NT patients.

## Background

Extranodal NK/T-cell lymphoma, nasal type (EN-NK/T-NT) is a major type of natural killer (NK) cell neoplasm, and its incidence is higher in Asia than it is in Western countries [1]. In our recent subtype distribution analysis of 142 Northern Chinese patients with peripheral NK/T cell lymphomas, EN-NK/T-NT was the most prevalent subtype (38.0%) [2]. This tumour usually presents with highly aggressive clinical progression, but the prognosis is variable and depends strongly on clinical factors. Our understanding of the pathological prognostic factors of this disease and the molecular characteristics of its pathogenesis remain limited.

In the last several decades, there has been extensive research on the development and molecular basis of EN-NK/T-NT implicating putative oncogenic mechanisms in its marked aggressiveness and poor survival. Results from gene expression profiling experiments suggest that the platelet-derived growth factor alpha, nuclear factor-κB, and the signal transducer and activator of transcription-3 signalling pathways may be involved in the angiogenesis, immunosuppression, proliferation, and survival of EN-NK/T-NT [3,4]. The overexpression of transcription factors and aberrant microRNAs (miRNAs) has also been associated with tumour oncogenesis [5-7].

Previous genome-wide studies have identified a deletion at 6q21 as the most frequent aberration in NK cell neoplasms [8-10]. Further detailed analysis suggests that positive regulatory domain containing I (PRDM1) is the most likely target gene in del6q21 [11]. The inactivation of PRDM1 has been detected in neoplastic NK cells, suggesting that it acts as a tumour suppressor gene [12]. However, the loss of PRDM1 is also associated with promoter CpG island hypermethylation in 71% of NK cell lymphomas [12]. Moreover, PRDM1 expression can be detected independent of the 6q21 deletion, and differences in the protein and mRNA levels of PRDM1 have been observed [3,11,13]. These results suggest a complex mechanism of PRDM1 inactivation in NK/T lymphomas.

In the present study, we investigated the expression of the PRDM1 protein in EN-NK/T-NT and the biological role of PRDM1 in the evaluation of the clinical outcome of EN-NK/T-NT patients. We also demonstrated a regulatory relationship between miR-223 and PRDM1, providing new insight into the inactivation of PRDM1 in EN-NK/T-NT.

## Materials and methods

### Patients and samples

A total of 61 cases of EN-NK/T-NT of the upper aerodigestive tract were retrieved from the Department of Pathology, Peking University First Hospital. The histological specimens were fixed in 10% buffered formalin and processed for routine paraffin-embedding. Histological sections with a thickness of 4 μm were stained with haematoxylin and eosin and used for immunoperoxidase procedures. EN-NK/T-NT was diagnosed based on combined morphological and immunophenotypical findings (including positive CD56 and cytotoxic proteins), as well as Epstein-Barr virus (EBV) positivity as determined by in situ hybridisation (ISH) with an EBV-encoded small RNA (EBER-1) probe, according to the WHO classification [14].

The 61 patients included 34 males and 27 females with ages ranging from 8 to 86 years (median 42 years). We obtained clinical information on all cases, and follow-up data for 35 patients. The follow-up period was defined as starting from the date of initial diagnosis to the patient’s death or last follow-up visit. Follow-up duration ranged from 1 to 120 months (median 20 months) for survivors.

The study was approved by the ethics committee of Peking University First Hospital (No. 2013[571]) and was performed according to ethics committee regulations and in compliance with the Declaration of Helsinki. The ethics committee of Peking University First Hospital specifically approved waiving the need for informed consent from participants because this was a retrospective study using archival surgical specimens with definitively established diagnoses. Only a few specimens were obtained for study to ensure the integrity of the remaining tissues. The patient data were obtained from the medical record library through a double-blind process and were analysed anonymously. There was no risk of conflict of interest for the patients.

### Cell lines and cell culture

We utilised three NK/T-cell lymphoma cell lines: YT [15], NKL [16], and NK92 [17]; the human chronic myelogenous leukaemia cell line K562; and the human embryonic kidney cell line 293 T. YT and NKL cells were obtained from Beijing Hong Bokang Biological Technology (Beijing, China). NK92, K562, and 293 T cells were purchased from the Chinese Academy of Medical Sciences (Beijing, China). YT, NKL, and K562 cells were cultured in RPMI medium 1640 (Invitrogen, Carlsbad, CA, USA) with 10% foetal bovine serum (Bio-Chrome, Germany). NK92 cells were maintained in Alpha Minimum Essential medium (Hyclone, UT, USA) with 12.5% horse serum and 12.5% foetal bovine serum. For NKL and NK92 cells, which are interleukin-2 (IL-2) dependent, the media were also supplemented with 100 U/mL human recombinant IL-2 (PeproTech, London, UK). 293 T cells were cultured in Dulbecco’s modified Eagle’s medium (Invitrogen, Carlsbad, CA, USA) with 10% foetal bovine serum.

### Immunohistochemistry

Immunohistochemistry (IHC) staining was performed using the DAKO EnVision detection kit (Dako, Glostrup, Denmark). The tissue sections were subjected to heat-induced antigen retrieval in EDTA buffer (pH 9.0). A primary antibody against PRDM1 (clone C14A4, Cell Signaling Technology, Beverly, MA, USA) was used. A positive nuclear staining pattern was interpreted as representing PRDM1 immunoreactivity. Based on Garcia and Nie’s investigations [18-20], positive expression of PRDM1 was defined as nuclear staining in 10% or more of the tumour population, and the stain grading was semi-quantitatively estimated as follows: negative (0% to <10%), weak (10% to ≤50% positive cells), or strong (>50% to 100% positive cells). Samples from plasma cell myelomas, tonsils, and the squamous epithelium of nasal mucosa were used as positive controls for PRDM1 staining. For the negative control reactions, Phosphate buffer saline (PBS) was used instead of the primary antibody.

### Quantitative real-time polymerase chain reaction for PRDM1α mRNA

We performed quantitative real-time polymerase chain reaction (qRT-PCR) to detect PRDM1α mRNA level. Total RNA was isolated from primary EN-NK/T-NT formalin-fixed paraffin-embedded (FFPE) tissues and cell lines (YT, NK92, NKL, and K562) using RNeasy FFPE kit (Qiagen, Crawley, UK) and mirVana miRNA isolation kit (Applied Biosystems, Foster City, CA, USA) according to the manufacturer’s instructions. A pathologist estimated the tumor region of the EN-NK/T-NT specimens on hematoxylin and eosin–stained slides. The concentration and quality of the total RNA was assessed with a NanoDrop 2000 spectrophotometer (Thermo Fisher Scientific, MA, USA). cDNA was synthesized from 1 μg of total RNA using random primers and AMV Reverse Transcriptase (Promega, Wisconsin, USA).

qRT-PCR assay for PRDM1α mRNA was performed using the Applied Biosystems Power SYBR Green PCR Master Mix and ABI-7300 real-time PCR system (Applied Biosystems, Foster City, CA, USA). The PCR reaction was conducted using 50 ng of cDNA template under the following conditions: 95°C for 10 min; 40 cycles at 95°C for 15 sec, 57°C for 30 sec, 72°C for 1 min. Primers for qRT-PCR assay are as follows: PRDM1: forward (5′-TCCAGCACTGTGAGGTTTCA-3′), reverse (5′-TCAAACTCAGCCTCTGTCCA-3′); β-actin: forward (5′-ATCATGTTTGAGACCTTCAACA-3′), reverse (5′-CATCTCTTGCTCGAAGTCCA-3′). The quantitative level of PRDM1α mRNA was normalized to β-actin using the cycle threshold (Ct) method (2^-△△Ct^ method). For each sample, 3 independent experiments were made with triplicates for each experiment. Samples from plasma cell myeloma and tonsil were used as positive controls for PRDM1α mRNA detection.

### ISH detection

ISH for miR-223, miR-886-3p, and miR-34c-5p was performed for 31 EN-NK/T-NTs, 10 peripheral T-cell lymphomas, and 13 inflammatory nasal mucosa specimens. The presence of NK cells within the inflammatory nasal mucosa specimens were identified by CD56 immunostaining. Probes labelled with a locked nuclear acid (LNA)™ probe for miR-223, miR-886-3p, and miR-34c-5p were designed and generated by Bio Perfectus Technologies (Jiang-su, China) according to sequences in the miRbase (Table 1).

**Table 1:** Sequences of in situ hybridisation probes for miR-223, miR-886-3p, and miR-34c-5p

**miRNA**	**MiRbase no.**	**Genomic location**	**Probe**
hsa-miR-223	MIMAT0000280	Xq12	5′-TGGGGTATTTGACAAACTGACA-3′
hsa-miR-886-3p	MIMAT0004906	5q31.1	5′-AAGGGTCAGTAAGCACCCGCG-3′
hsa-miR-34c-5p	MIMAT0000686	11q23.1	5′-GCAATCAGCTAACTACACTGCCT-3′

The ISH assays for miRNAs were performed as follows: FFPE tissues were routinely deparaffinised in xylene and rehydrated with an ethanol gradient, treated with 1 mg/ml Proteinase K for 10 min at 37°C, fixed with 4% formaldehyde for 10 min, and then dehydrated in ice-cold 90% ethanol. A 20-μL volume of hybridisation mixture consisting of 2 μL of the indicated LNA™ probe and 18 μL of a solution of 200 μg/mL salmon sperm DNA, 1 mg/mL dithiothreitol (DTT), 50% formamide, 2× Denhardt’s, 1 mg/mL polyglucosan, and 2× saline-sodium citrate (2× SSC) was applied to each slide. The hybridisation reactions were performed overnight at 42°C in a humidified chamber. The sections were stringently rinsed 3 times for 15 min each in 2× SSC at 37°C, and endogenous peroxidases were blocked with 10% H_2_O_2_ for 20 min. After 2 washes in 1× PBS for 10 min, the slides were blocked with goat serum (1:100) for 30 min. The slides were then incubated with mouse anti-digoxin antibody for 20 h at 4°C. The slides were washed twice with 1× PBS, incubated with polymer auxiliary agent for 30 min, and washed with 1× PBS for 10 min. Goat anti-mouse secondary antibody was added to the slides. After 2 washes with 1× PBS, DAB staining was performed. miR-223-, miR-886-3p-, or miR-34c-5p-positive EN-NK/T-NT tissue was used as a positive control for miR-223, miR-886-3p, or miR-34c-5p staining, respectively. For negative control samples, the hybridisation reactions were performed with a sense probe. Cytoplasmic staining was interpreted as miRNA expression, and positive expression was defined as staining of 10% or more of the cells in each tumour. The grading was semi-quantitatively estimated as follows: negative (0% to <10%), weak (10% to ≤50% positive cells), or strong (>50% to 100% positive cells).

### miRNA detection

To quantify miRNA levels in cell lines, qRT-PCR was performed using the Taqman MicroRNA RT Kit, Taqman MicroRNA Assays, and TaqMan Universal Master Mix II (Applied Biosystems, Foster City, CA, USA) in an Applied Biosystems ABI-7300 real-time PCR system according to the manufacturers’ recommendations. The expression level of U6 RNA was used as an internal control for normalisation. The expression level of the indicated miRNA relative to U6 was defined using the Ct method. Relative quantification using the 2^-△△Ct^ method was performed for each miRNA. We maintained an RNase-free work environment during all protocols and utilised diethylpyrocarbonate (DEPC)-treated water to prepare all solutions.

### Prediction of miRNA target genes

We predicted miRNA target genes using online prediction algorithms, including Target Scan Human 6.0 (http://www.targetscan.org/vert_60), PICTAR-VERT (http://pictar.mdc-berlin.de/cgi-bin/PicTar_vertebrate.cgi), MICRORNA.ORG (http://www.microrna.org/microrna/getMirnaForm.do), and DIANA-MICROT (http://diana.cslab.ece.ntua.gr/micro-CDS).

### Plasmid construction

The 3′-untranslated region (UTR) of human PRDM1 mRNA, which contains 3 putative miRNA target sites, was PCR amplified from human genomic DNA using the forward primer 5′-ATCGAGCTCAATCACGTCGGTATGATTGG-3′ and the reverse primer 5′-ACGCGTCGACAGTTTGTTGTTCTAGCAAAGTA-3′ and subsequently cloned into the pmirGLO Dual-Luciferase miRNA Target Expression Vector (Promega, Wisconsin, USA) using the *Sac*I and *Sal*I restriction sites to generate the wild-type reporter vector PRDM1 3′-UTR.

Mutant reporter constructs were generated via the QuikChange Site-Directed Mutagenesis Kit (Stratagene, La Jolla, CA, USA) to generate 2 consecutive nucleotide substitutions at the centre of each putative miR-223 binding site. The 3 putative binding sites in the PRDM1 3′-UTR were numbered 1 to 3 according to their positions from the distal to proximal end. The 3 putative binding sites were mutated individually or in combination as follows: Mut1, Mut2, Mut3, Mut1 + 2, Mut1 + 3, Mut2 + 3, and Mut1 + 2 + 3. The following primers were used (mutant nucleotides indicated in bold): Mut1: 5′-CACAGAAATAAAAAA**GA**GACTTTACCGCTGC-3′; Mut2: 5′-CTGTAACTTCCAAGAC**AC**ACAGCTTTTTATGTATC-3′; and Mut3: 5′-CTACTCAAAGTTAAAA**GA**GACCAAAGTTACTGGC-3′. All constructs were verified by sequencing.

### Luciferase assays

For luciferase assays, 293 T cells were transiently co-transfected with 150 ng of each of the reporter constructs (wild-type and mutant pmirGLO Dual-Luciferase miRNA Target Expression Vector expressing both firefly and renilla luciferase) and 8 pmol of mirVana miRNA Mimic-223 or mirVana miRNA Mimic Negative Control (Ambion, Austin, TX) in 24-well plates using Lipofectamine™ 2000 (Invitrogen, Carlsbad, CA, USA).

We analysed luciferase activity in the cells at 24 h after co-transfection using the Dual-Glo® Reporter Assay System (Cat. # E1910, Promega, Wisconsin, USA) and a Wallac Microbeta Trilux detector (Perkin Elmer, MA, USA). The normalised firefly luciferase activity (firefly luciferase activity/renilla luciferase activity) of each construct was compared to that of the pmirGLO Vector control. For each transfection, the average luciferase activity from 4 independent experiments is reported.

### Transfection assays and western blot

For electroporation, 2 × 10^6^ YT cells were resuspended in 300 μL RPMI 1640 medium without serum or antibiotics and mixed with 150 pmol mirVana miRNA Mimic-223 or mirVana miRNA Mimic Negative Control. Electroporation was performed with a BTX ECM 830 electroporator (BTX, San Diego, CA, USA) with a single pulse of 120 V and 20 ms. After transfection, the cells were immediately transferred to an incubator at 37°C and incubated for 5 min. The transiently transfected cells were then cultured in pre-warmed complete RPMI 1640 medium. The cell viability was monitored by microscopic observation. The cells were collected at 24 h and 48 h after electroporation and subjected to total RNA isolation and western blot detection, respectively. The transfection efficiency was evaluated by detecting the fold increase of miR-223 using qRT-PCR.

In addition, we transiently transfected 2.5 × 10^5^ NK92, NKL, or K562 cells with 150 pmol of mirVana miR-223 inhibitor (Ambion, Austin, TX) using HiPerFect Transfection Reagent (Qiagen, Valencia, CA, USA) according to the manufacturer’s instructions. Transfection with the mirVana miRNA Mimic Negative Control (Ambion, Austin, TX) was used as a negative control. We collected NK92, NKL, or K562 cells at 24 h and 48 h after transfection for total RNA isolation and western blot detection, respectively. The detection of the fold decrease of miR-223 in cells was performed to estimate the transfection efficiency by qRT-PCR.

Whole-cell lysates of transfected YT, NK92, NKL, or K562 cells were separated by electrophoresis in 10% sodium dodecyl sulphate polyacrylamide gels. The gels were electroblotted to polyvinylidene difluoride membranes (Millipore), and the membranes were then blocked with 5% skim milk for 1 h at room temperature, followed by incubation with a rabbit or mouse monoclonal antibody against PRDM1 (PRDI-BF1) (1:1,000; Cell Signaling Technology, Beverly, MA, USA) or β-actin (1:5,000; Roche Applied Science, Indianapolis, USA) overnight at 4°C. Horseradish peroxidase-conjugated secondary antibodies included anti-rabbit (1:5,000, Zhongshan, China) and anti-mouse (1:5,000, Zhongshan, China). PRDM1 expression was quantified by densitometry and normalised to β-actin.

### Semi-quantitative RT-PCR

A total of 1 μg of total RNA from electroporated YT cells was used to synthesise cDNA using AMV Reverse Transcriptase (Promega, Wisconsin, USA). We assessed the level of PRDM1 expression using the β-actin gene as an internal control. The primers of PRDM1α and β-actin for RT-PCR were described as above. The PCR conditions were as follows: 94°C for 3 min; 35 cycles at 94°C for 30 sec, 57°C for 30 sec, 72°C for 30 sec; and a final extension at 72°C for 5 min. The expected fragment sizes were 104 bp for PRDM1 and 318 bp for β-actin. We analysed the reactions using agarose gel electrophoresis.

### Statistical analysis

We used the Mann–Whitney *U*-test or Student’s *t*-test to analyse differential miRNA expression as determined by qRT-PCR miRNA assays and western blot result, and we estimated the statistical significance of the level of miRNA expression as determined by ISH using a *χ*^2^ test or Fisher’s exact test. The Spearman rank correlation coefficient test was utilised to correlate the expression of PRDM1 and miR-223. Treatment outcomes were measured by failure-free survival (FFS) and overall survival (OS). FFS was defined as the time from initial diagnosis to progression, relapse, or death from any cause. OS was calculated as the time from initial diagnosis to death from any cause or to last follow-up. The estimates of FFS and OS were calculated using the Kaplan-Meier method and compared to log-rank tests and multivariate analysis (Cox model). Differences were considered statistically significant when the 2-sided *P* value was less than 0.05. All analyses were performed using SPSS (Statistical Package for the Social Sciences) 13.0 software (Chicago, IL).

## Results

### Evaluation of PRDM1 expression in EN-NK/T-NT samples by IHC

**Figure 1:**
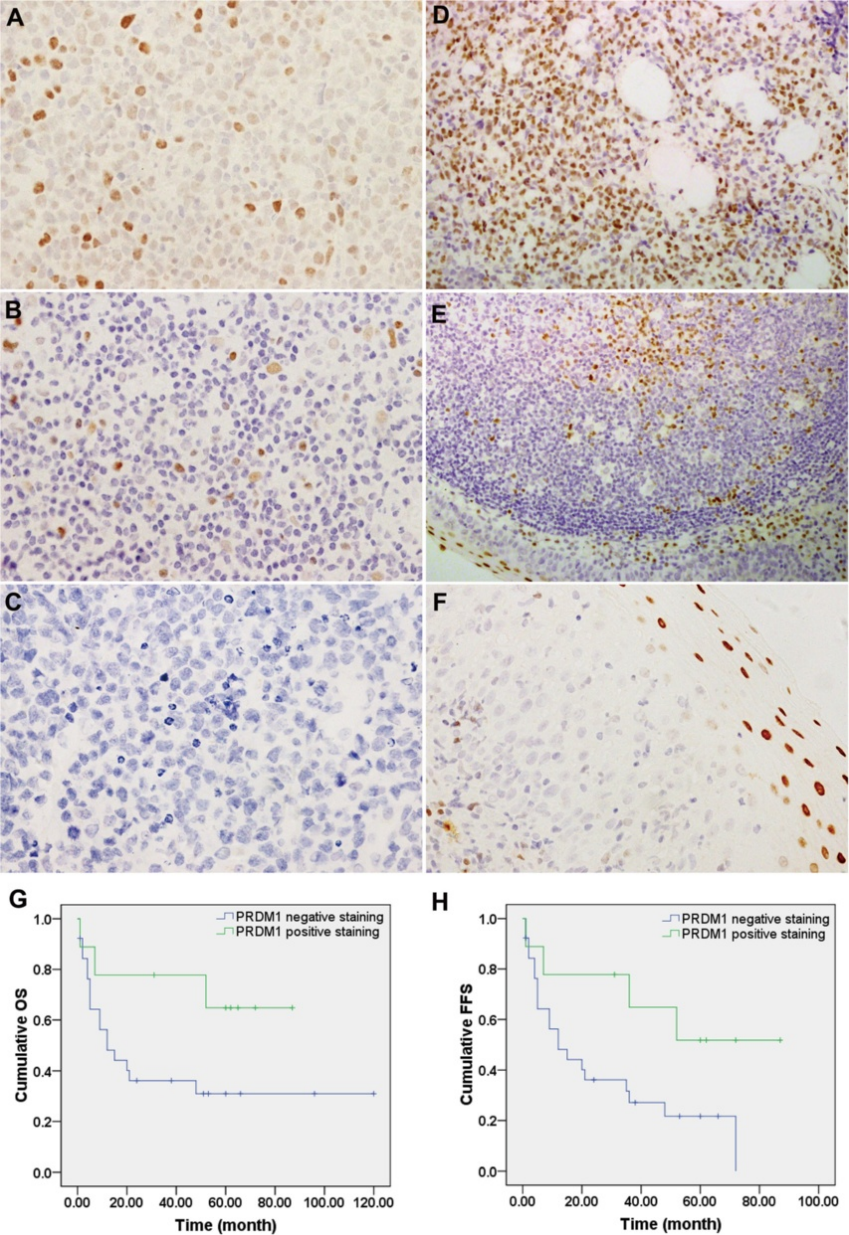
**Immunohistochemistry (IHC) and prognostic analysis of PRDM1 in extranodal NK/T-cell lymphoma, nasal type (EN-NK/T-NT) cases.** Examples of IHC analysis of PRDM1 in EN-NK/T-NT specimens and control samples. **(A)** PRDM1 staining in the nuclei of tumour cells was observed in approximately 50% of tumour cells in 1 case of EN-NK/T-NT; most cells had moderate to weak nuclear staining. **(B)** PRDM1 was expressed in approximately 10% of tumour cells in 1 case of EN-NK/T-NT. **(C)** No PRDM1 staining was detected in 1 case of EN-NK/T-NT. In the control cases, strong nuclear PRDM1 immunostaining was observed in plasma cell myeloma **(D)**, the epithelium and germinal centre of the tonsil **(E)**, and the squamous epithelium of the nasal mucosa **(F)** (all by IHC; A, B, C, and F are shown at 400× magnification; D and E are shown at 200× magnification). **(G)** and **(H)** Kaplan-Meier survival analysis demonstrated that PRDM1 expression predicted a favourable effect on overall survival (OS) and failure-free survival (FFS) of EN-NK/T-NT patients (*P* = 0.084 and *P* = 0.042, respectively).

The expression of PRDM1 protein in 61 primary EN-NK/T-NT tumour specimens was assessed by IHC. As shown in Figure 1A and B, PRDM1 positive staining was observed in the nuclei of tumour cells. The expression of PRDM1 was negative in the majority of EN-NK/T-NT samples (46/61, 75.41%) (Figure 1C), and the remaining EN-NK/T-NT cases (15/61, 24.59%) showed only weak staining (10%-50% positive cells) for PRDM1 (Figure 1A, B); no EN-NK/T-NT samples were strongly positive for PRDM1. By contrast, strong positive staining was observed in all the positive control cases, including samples from plasma cell myeloma (Figure 1D), tonsil (Figure 1E), and the squamous epithelium of nasal mucosa (Figure 1F); more than 50% of the tumour cells in these samples showed nuclear staining, and the staining intensity of the positive cells was distinctly stronger than that of the EN-NK/T-NT cases. Thus, these results demonstrate that PRDM1 protein expression is downregulated in EN-NK/T-NT cases, similar to results from a previous article [18].

### Correlation between PRDM1 expression and the clinical factors of EN-NK/T-NT patients

To identify the possible biological role of PRDM1 expression in EN-NK/T-NT, we analysed the correlation between the expression of PRDM1 and clinical findings in EN-NK/T-NT patients. Follow-up study of 35 cases showed mean and median survival periods of 32 months and 20 months, respectively. The 5-year OS rate was 37.14%. The clinical characteristics of the patients including sex, age, Ann Arbor Stage and patient outcome, and the results of the statistical analysis are summarised in Table 2.

**Table 2:** Correlation of PRDM1 and miR-223 expression with clinical factors and prognostic value

			**PRDM1 expression**					**miR-223 expression**		
	**n**	**Percent**	**Negative**	**Positive**	***P***	**n**	**Percent**	**Negative**	**Positive**	***P***
**Patients**	61					31				
male	34	55.74	26	8	0.829	19	61.29	5	14	0.704
female	27	44.26	20	7		12	38.71	4	8	
**Age (year)**	61					31				
<40	29	47.54	21	8	0.463	13	41.94	4	9	NA^※^
40-60	20	32.79	17	3		11	35.48	2	9	
>60	12	19.67	8	4		7	22.58	2	5	
**Stage**^**∆**^	46					26				
І/ІІ	18	39.13	9	9	0.009	9	34.62	3	6	0.661
III/IV	28	60.87	24	4		17	65.38	4	13	
**Outcome**	35					21				
alive	12	34.29	6	6	0.038	8	38.10	3	5	0.325
dead	23	65.71	20	3		13	61.90	2	11	
**5-year OS**	35					21				
Mean ± SD			39.49 ± 9.62	64.02 ± 11.48	0.045			53.40 ± 18.41	45.70 ± 10.05	0.504
**OS**	35					21				
Mean ± SD			44.72 ± 10.41	64.02 ± 11.48	0.084			53.40 ± 18.41	52.84 ± 10.70	0.784
**FFS**	35					21				
Mean ± SD			26.50 ± 5.60	57.41 ± 11.60	0.042			43.20 ± 16.89	38.99 ± 7.84	0.691

^※^NA, not analyzed, because of limited sample size.

^△^Ann Arbor Stage.

A univariate analysis of advanced stage (III/IV) disease showed significantly downregulated expression levels of PRDM1 (*P* = 0.009, Table 2). As expectedly, the frequency of PRDM1 expression distribution was significantly different among living and deceased patients (*P* = 0.038) and had a significant effect on the 5-year OS (*P* = 0.045). Notably, Kaplan-Meier single-factor analysis and the log-rank test revealed that PRDM1-positive staining predicted a favourable effect on OS and FFS (Table 2, Figure 1G and H), suggesting that the expression of PRDM1 may be an important predictive factor in EN-NK/T-NT patients. In addition, multivariate analysis and Cox regression combining Ann Arbor Stage revealed that PRDM1 expression status did not reach statistical significance as an independent predictor of 5-year OS (*P* = 0.556) and FFS (*P* = 0.727), but Ann Arbor Stage was an independent predictor of 5-year OS (*P* = 0.002) and FFS (*P* = 0.003).

### Detection of PRDM1 transcripts in EN-NK/T-NT

**Figure 2:**
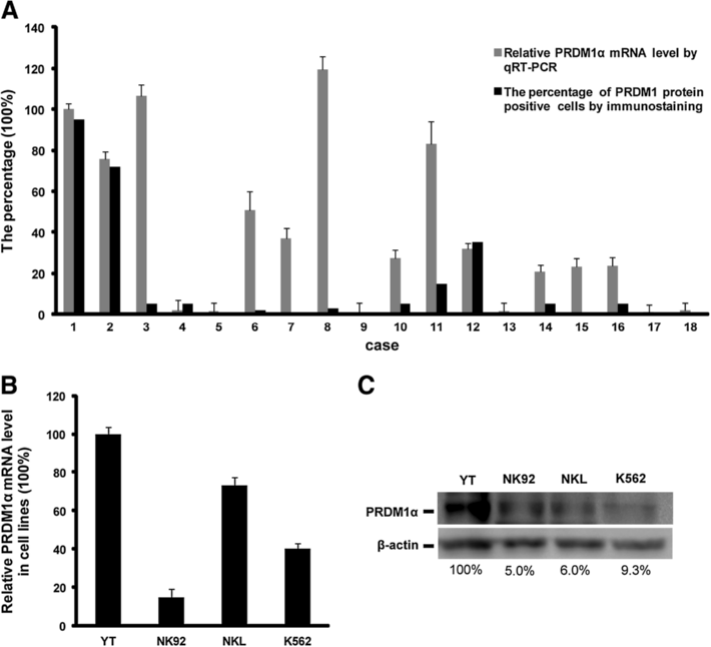
**Discrepancy between PRDM1α mRNA and protein expression in extranodal NK/T-cell lymphoma, nasal type (EN-NK/T-NT). (A)** The relative levels of PRDM1α mRNA by qRT-PCR and the corresponding PRDM1 protein by immunohistochemistry (IHC) were analysed in 16 EN-NK/T-NT cases, one plasma cell myeloma, and one tonsil case. Case #1 is plasma cell myeloma. Case #2 is tonsil, and cases #3 to #18 are 16 EN-NK/T-NT cases. Levels of PRDM1α mRNA in the tonsil and EN-NK/T-NT cases were estimated relative to that in plasma cell myeloma (arbitrarily set as 100%), which showed strong expression of PRDM1 protein. The data of PRDM1α mRNA by qRT-PCR are presented as mean ± SE of 3 independent experiments. Expression of PRDM1 protein in formalin-fixed paraffin-embedded sections of EN-NK/T-NT specimens, plasma cell myeloma, and one tonsil case was determined by immunostaining and assessed by the percentage of PRDM1 positive cells. Of 16 EN-NK/T-NT cases, 9 cases (#3, 6, 7, 8, 10, 11, 14, 15, and 16) showed high level of PRDM1α mRNA relative to plasma cell myeloma by qRT-PCR but low or absent percentage of PRDM1 protein positive tumor cells by IHC. **(B)** PRDM1α mRNA was determined by qRT-PCR in NK/T-cell lymphoma cell lines YT, NK92, and NKL, and the human chronic myelogenous leukaemia cell line K562 (mean ± SE of 3 independent experiments). The level of PRDM1α transcript was assessed relative to that in YT cells (arbitrarily considered as 100%). PRDM1α mRNA levels in NK92, NKL, and K562 cells were 15.0%, 73.0%, and 40.1% of those in YT cells, respectively. **(C)** The expression of PRDM1α protein was detected in cell lines by western blot. The density of PRDM1α in YT cells was set as 100%, and the levels in NK92, NKL, and K562 cells were calculated as 5.0%, 6.0%, and 9.3% of YT cells, respectively.

As mentioned earlier in the Background, the inactivation of PRDM1 cannot be attributed only to 6q21 deletion. Epigenetic regulation such as translational suppression or DNA methylation may also be involved [12]. To further clarify the molecular mechanism of PRDM1 inactivation, we compared the PRDM1 protein expression with the PRDM1 transcript level in EN-NK/T-NT specimens and NK/T-cell lymphoma cell lines. As shown in Figure 2A, we set plasma cell myeloma (case #1) as having strong expression of PRDM1 protein as 100%. Case #2 indicates tonsil, as a control with relative high percentage of PRDM1 protein positive cells. Case #3 to #18 indicates 16 EN-NK/T-NT cases. We observed the discordance between PRDM1 transcript and protein expression in most EN-NK/T-NT cases (9/16, 56.25%) (Figure 2A). High level of PRDM1α mRNA relative to plasma cell myeloma and tonsil was detected in 9 EN-NK/T-NT cases (#3, 6, 7, 8, 10, 11, 14, 15, and 16) by qRT-PCR, but the percentage of PRDM1 positive tumor cells was low or absent in IHC, indicating that the degree of PRDM1 transcript did not translate to the same extent as PRDM1 protein. These findings suggest that the decreased PRDM1 protein may be associated with post-transcriptional regulation.

Similarly, both qRT-PCR and western blot analysis revealed the discrepancy between PRDM1 transcript and its protein in some NK/T-cell lymphoma cell lines. As shown in Figure 2B and Figure 2C, in contrast to YT or NK92 cells, which presented consistent levels in both transcription and protein of PRDM1, PRDM1 transcripts in NKL cells are estimated at about 73.0% of those in YT cells (Figure 2B), whereas PRDM1α protein is just 6.0% (Figure 2C). Similarly, PRDM1α transcript and protein levels in K562 cells, the human chronic myelogenous leukaemia cell line, are 40.1% and 9.3% of YT cells, respectively (Figure 2B, C). Therefore, what we have observed in EN-NK/T-NT tissues and cell lines strongly imply the possibility that post-transcriptional regulation may abrogate the PRDM1 protein expression.

### Altered miRNA expression in EN-NK/T-NT lymphoma

miRNAs are a novel class of non-coding small RNAs that negatively regulate protein expression via specific binding to their target sites in the 3′-UTR of their target mRNAs, initiating a translational blockade or the degradation of target mRNAs. We have previously confirmed the upregulation of miR-223 and miR-886-3p and the downregulation of miR-34c-5p in EN-NK/T-NT cases; these changes are significantly different from those occurring in inflammatory nasal mucosa based on global miRNA expression profiling and qRT-PCR miRNA assays [21]. We hypothesised that in addition to the frequent deletions and DNA methylation reported previously, aberrant miRNAs may be responsible for the downregulation of the PRDM1 protein in EN-NK/T-NT.

**Figure 3:**
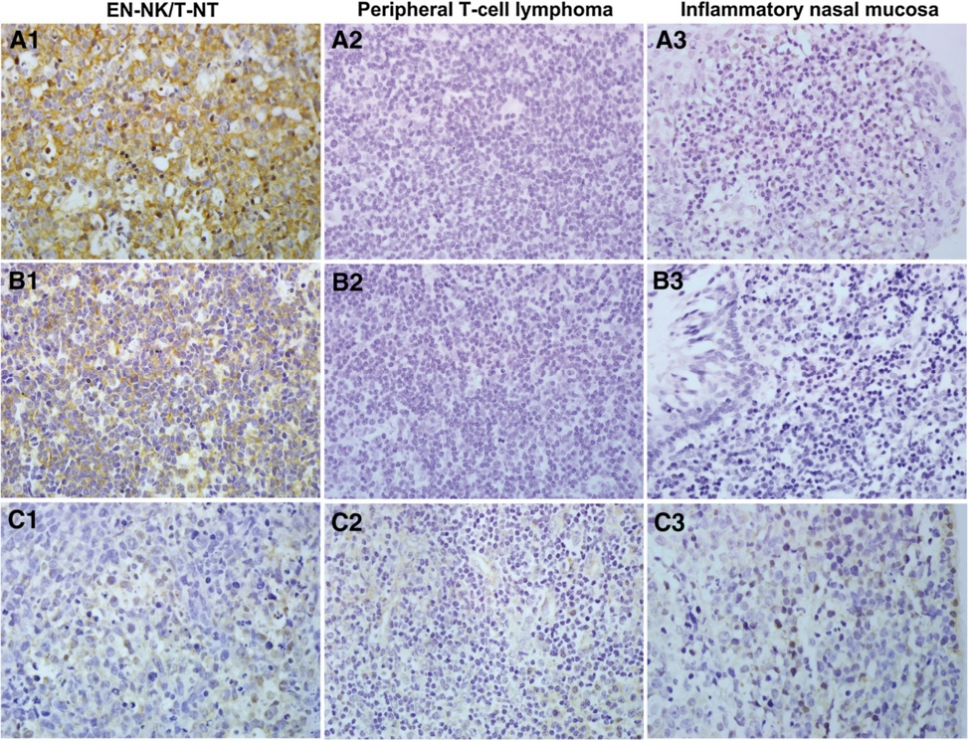
**Representative cases of miRNA expression identified by in situ hybridisation (ISH).** ISH analysis revealed characteristic upregulation of miR-223 in the cytoplasm of extranodal NK/T-cell lymphoma, nasal type (EN-NK/T-NT) tumour cells **(A1)**, whereas no signal was detected in peripheral T-cell lymphoma **(A2)** and inflammatory nasal mucosa **(A3)**. In addition, miR-886-3p was also overexpressed in the cytoplasm of EN-NK/T-NT tumour cells **(B1)** but was negative in peripheral T-cell lymphoma **(B2)** and inflammatory nasal mucosa **(B3)**. There were no miR-34c-5p signals in EN-NK/T-NT samples **(C1)**, peripheral T-cell lymphoma samples **(C2)**, or inflammatory nasal mucosa samples **(C3)**. All images show ISH at 400x magnification.

**Figure 4:**
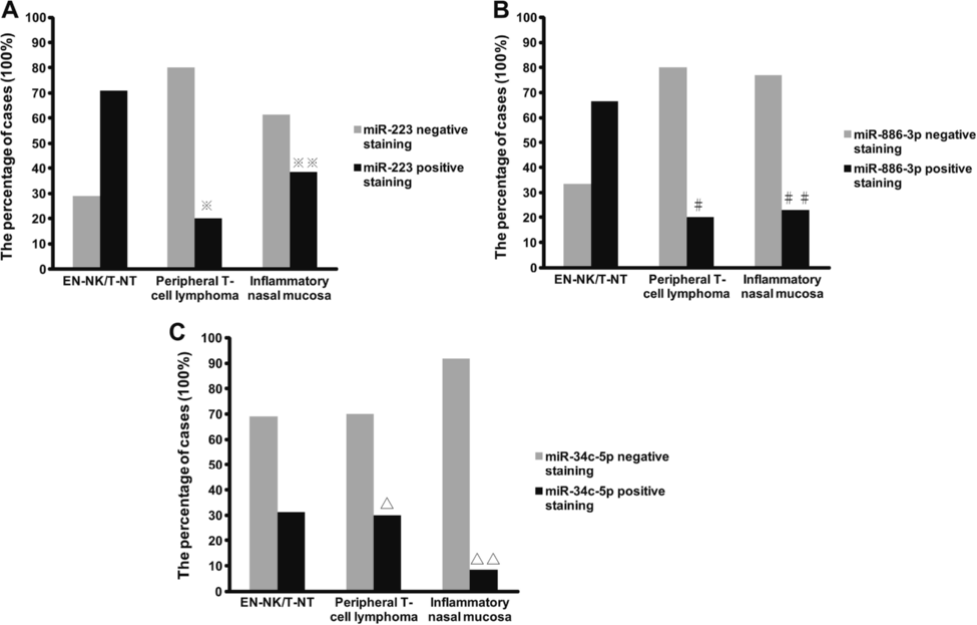
**Statistical analysis of miR-223, miR-886-3p, and miR-34c-5p expression by in situ hybridisation (ISH).** The expression percentage of miR-223, miR-886-3p, and miR-34c-5p was statistically analysed in extranodal NK/T-cell lymphoma, nasal type (EN-NK/T-NT), peripheral T-cell lymphoma and inflammatory nasal mucosa cases by ISH. Statistically, ISH results revealed that the expression level of miR-223 was significantly higher in EN-NK/T-NT cases than in peripheral T-cell lymphoma **(A**, ^※^*P* = 0.013) and in inflammatory nasal mucosa **(A**, ^※※^*P* = 0.043). Similarly, miR-886-3p expression upregulated in EN-NK/T-NT cases compared to peripheral T-cell lymphoma **(B**, ^#^*P* = 0.028) and inflammatory nasal mucosa **(B**, ^##^*P* = 0.022). However, the expression level of miR-34c-5p **(C**, ^∆^*P* = 1.000 and ^∆∆^*P* = 0.254) did not differ significantly between these 3 groups.

Because of the highly inflammatory background of EN-NK/T-NT, we used ISH to determine the expression status of miR-223, miR-886-3p, and miR-34c-5p in tumour cells. ISH analysis of FFPE tissues from EN-NK/T-NT demonstrated strong expression of miR-223 and miR-886-3p in the cytoplasm of EN-NK/T-NT tumour cells and weak to no staining in peripheral T-cell lymphoma or inflammatory nasal mucosa; miR-34c-5p staining was weak in most samples from these 3 groups. Representative ISH results for miR-223, miR-886-3p, and miR-34c-5p are shown in Figure 3. As shown in Figure 4A, the expression of miR-223 was statistically greater in EN-NK/T-NT cancer cells than in peripheral T-cell lymphoma (*P* = 0.013) and inflammatory nasal mucosa samples (*P* = 0.043). In addition, miR-886-3p also upregulated in EN-NK/T-NT samples, which was significantly different from peripheral T-cell lymphoma (*P* = 0.028) and inflammatory nasal mucosa samples (*P* = 0.022) (Figure 4B). Nevertheless, miR-34c-5p expression showed no significant difference between primary EN-NK/T-NT, peripheral T-cell lymphoma, and inflammatory nasal mucosa tissues (*P* = 1.000 and *P* = 0.254, respectively) (Figure 4C). In addition, the ISH results of miR-223, miR-886-3p, and miR-34c-5p were cross-validated with qRT-PCR results in 15 EN-NK/T-NT FFPE cases. Both the ISH results and the previous qRT-PCR microRNA assays were highly consistent in the EN-NK/T-NT cases (91.67% consistency for miR-223, 75.00% for miR-886-3p, and 58.33% for miR-34c-5p), which is further evidence of the aberrant overexpression of miR-223 and miR-886-3p. Thus, the upregulation of miR-223 and miR-886-3p might be involved in the oncogenesis of EN-NK/T-NT and associated with PRDM1 inactivation.

### Bioinformatic prediction of potential miRNA target genes

**Figure 5:**
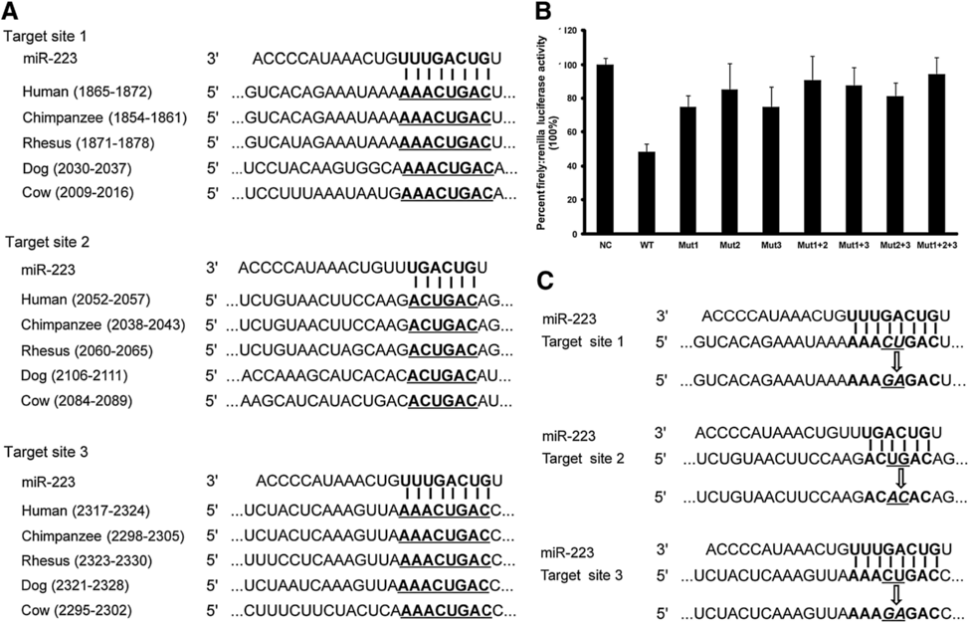
**Verification of PRDM1 as a direct target gene of miR-223. (A)** The complementarity between miR-223 and its 3 conserved putative binding sites in the PRDM1 3′-untranslated region (UTR) is highlighted in bold between different species. **(B)** Luciferase reporter assays were performed in 293 T cells that were co-transfected with miR-223 mimic and wild type pmirGLO expression-PRDM1-3′UTR (WT) reporter plasmid or mutant pmirGLO expression-PRDM1-3′UTR reporter plasmids harbouring point mutations in the target sites for miR-223 (Mut1, Mut2, Mut3, Mut1 + 2, Mut1 + 3, Mut2 + 3, and Mut1 + 2 + 3). Mimic Negative Control was used as a negative control (NC). Firefly luciferase activity was normalised relative to Renilla luciferase activity. Transfection of the miR-223 mimic resulted in a marked decrease in luciferase activity in the WT group compared to the NC group (48.08%). Mutations in each of the putative target sites or combined mutations restored luciferase activity to varying degrees: 74.87% for Mut1, 85.21% for Mut2, 74.84% for Mut3, 90.76% for Mut1 + 2, 87.55% for Mut1 + 3, 81.15% for Mut2 + 3, and 94.51% for Mut1 + 2 + 3. Data are presented as mean ± SE of 4 independent experiments. **(C)** Two nucleotides in the middle of each target site were mutated to generate different mutant luciferase reporters.

To identify potential miRNA:mRNA target interactions, we utilised bioinformatics prediction algorithms including Target Scan Human 6.0, PICTAR-VERT, MICRORNA. ORG, and DIANA-MICROT. Bioinformatics prediction algorithms did not predict a target interaction between miR-886-3p and PRDM1 mRNA. Notably, 3 putative miR-223 binding sites were predicted in the 3'-UTR of the PRDM1 mRNA (Figure 5A). Moreover, the bases required for efficient pairing between the 5'-end sequence, also known as the “seed sequence”, of miR-223 and the complementary sequences of PRDM1 3′-UTR are evolutionarily conserved (Figure 5A), suggesting a potential regulatory role of miR-223 for PRDM1 expression.

### The expression of PRDM1 in EN-NK/T-NT correlates with miR-223

**Figure 6:**
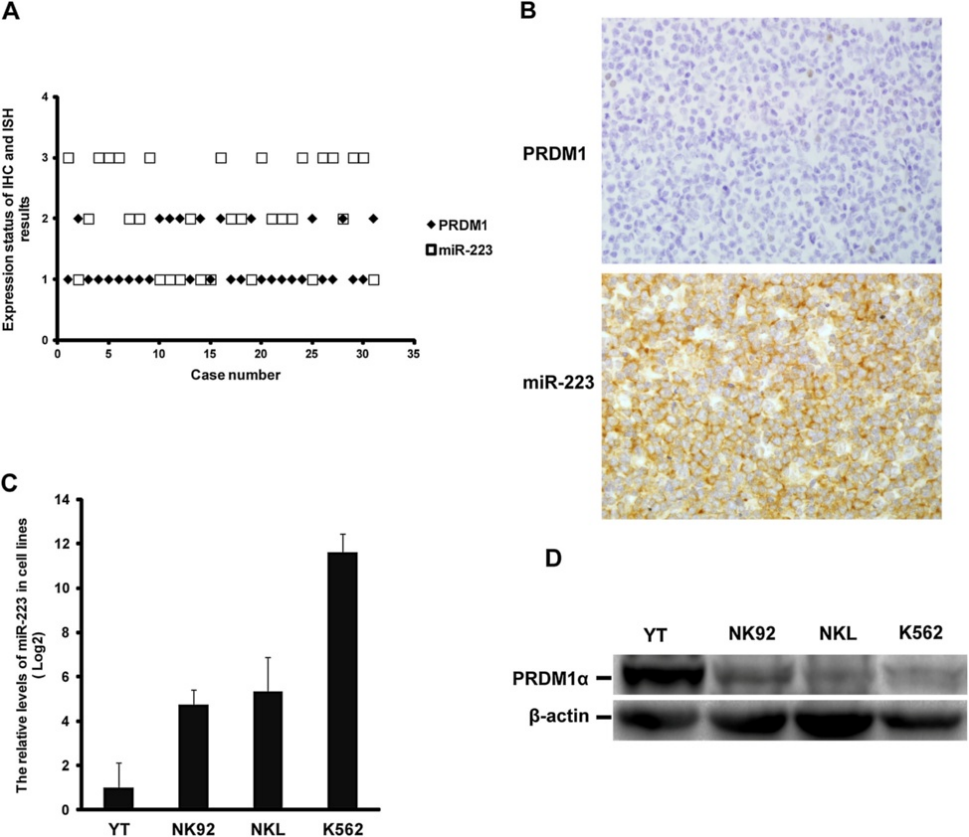
**Correlation of the expression of PRDM1 and miR-223 in extranodal NK/T-cell lymphoma, nasal type (EN-NK/T-NT). (A)** The expression of PRDM1 and miR-223 in EN-NK/T-NT cases were analysed by immunohistochemistry (IHC) and in situ hybridisation (ISH), respectively, and the result is shown as a scatter diagram. As described in the Materials and Methods section, these results were semi-quantitatively scored into 3 grades according to the number of positive tumour cells. In this figure, the numbers of ordinate are as follows: “1” indicates negative (0% to <10% positive cells), “2” indicates weak (10% to ≤50% positive cells), and “3” indicates strong (>50% to 100% positive cells). Statistically, a significantly opposing correlation was observed between the levels of PRDM1 protein and miR-223 expression in 31 EN-NK/T-NT cases (*P* < 0.001); only 2 cases had the same relative expression levels of PRDM1 and miR-223. **(B)** One representative case of EN-NK/T-NT was negative for PRDM1 by IHC but strongly positive for miR-223 by ISH (400×). **(C)** qRT-PCR analysis revealed much lower levels of miR-223 in YT cells than in NK92, NKL, and K562 cells (mean ± SE of 3 independent experiments). **(D)** Western blotting revealed markedly higher levels of PRDM1α protein in YT cells than in NK92, NKL, and K562 cells.

To investigate the association between PRDM1 and miR-223 in EN-NK/T-NT cases, we performed a correlative analysis between PRDM1 immunostaining and miR-223 ISH. As shown in the scatter diagram (Figure 6A), there is a significant inverse correlation between the levels of PRDM1 expression and miR-223 expression in EN-NK/T-NT cases (*P* < 0.001). Only 2 cases exhibited similar expression levels of miR-223 and PRDM1. Figure 6B shows one representative case of this inverse correlation in which ISH revealed strong positive expression of miR-223, and IHC indicated no PRDM1 expression in EN-NK/T-NT.

In addition, we assessed the correlation between PRDM1 and miR-223 using qRT-PCR and western blot analysis of 3 NK/T lymphoma cell lines: YT, NK92, and NKL. Since K562 cells have a high level of miR-223 but lack PRDM1 expression, we used this as a control cell line. The level of miR-223 was much lower in YT cells than in NK92 and NKL cells (Figure 6C), and conversely, PRDM1α protein was markedly higher in YT cells than in NK92 and NKL cells (Figure 6D).

Taken together, these results demonstrate an opposing expression pattern of PRDM1 protein and miR-223 in primary EN-NK/T-NT tissues or in cultured NK/T lymphoma cells, suggesting that miR-223 might regulate the expression of PRDM1.

### Identification of PRDM1 as a direct target gene of miR-223

To identify PRDM1 3′-UTR as a direct target gene of miR-223, we constructed a luciferase reporter plasmid containing the PRDM1 3′-UTR by inserting the 3 predicted target sequences into the pmirGLO expression vector. qRT-PCR analysis revealed that miR-223 is not endogenously expressed in 293 T cells. Thus, luciferase reporter assays were performed with 293 T cells by co-transfecting pmirGLO Expression-PRDM1-3′UTR with mirVana miRNA Mimic-223 (WT group) or Mimic Negative Control (NC group). The luciferase activity of the WT group decreased to 48.08% upon the ectopic expression of miR-223 compared to the NC group (Figure 5B), demonstrating the direct effect of miR-223 on the PRDM1 3′-UTR.

To clarify the interaction between miR-223 and its predicted target sequences, a panel of reporter constructs containing individual or combined mutations in the predicted target sequences was generated as shown in Figure 5C. Each of these reporters was individually transfected into 293 T cells with the miR-223 mimic. Mutagenesis effectively restored luciferase activity to varying degrees (74.87% for Mut1, 85.21% for Mut2, and 74.84% for Mut3, Figure 5B). Moreover, the combined mutation of any 2 target sites induced an increased restoration of luciferase activity (90.76% for Mut1 + 2, 87.55% for Mut1 + 3, and 81.15% for Mut2 + 3, Figure 5B). Notably, the repression of luciferase activity by miR-223 was nearly eliminated (94.51%) when all 3 predicted target sites were mutated (Figure 5B). In addition, luciferase activity recovered more strongly with the mutation of target site 2 compared to mutations of the other 2 target sites, implying that target site 2 may play a more important role in the direct binding between miR-223 and the PRDM1 3′ -UTR.

Taken together, this experimental evidence demonstrates that the 3 predicted target sites in the PRDM1 3′-UTR all contribute to the direct post-transcriptional regulation of PRDM1 expression by miR-223, and that a differential and cooperative effect exists between these 3 putative binding sites.

### Ectopic expression of miR-223 leads to the downregulation of the PRDM1 protein

**Figure 7:**
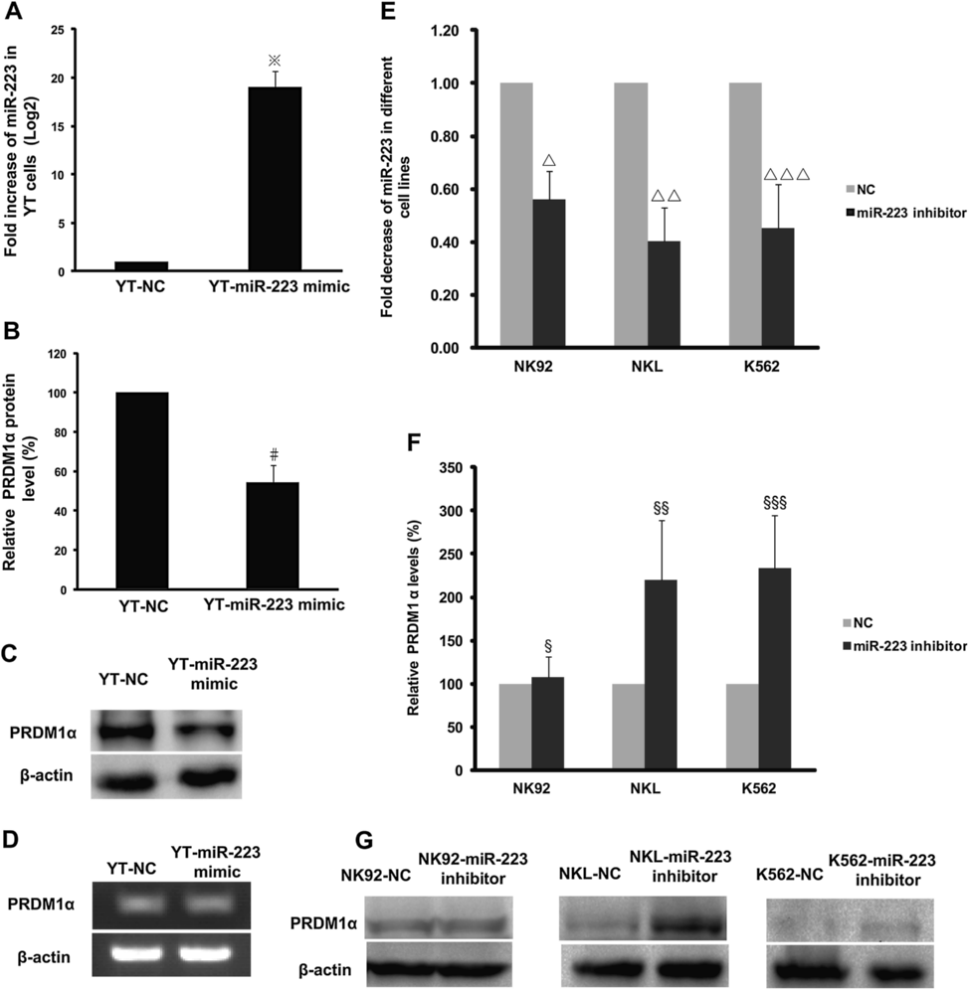
**Endogenous PRDM1 protein expression is affected by increased miR-223 or decreased miR-223.** A miR-223 mimic or mimic negative control (NC) was transfected into YT cells by electroporation. **(A)** qRT-PCR analysis revealed a significantly increased level of miR-223 in YT cells transfected with miR-223 mimic compared to NC. The results were confirmed in 3 independent experiments with data presented as mean ± SE (^※^*P* < 0.001). **(B)** Western blot showed that PRDM1α protein level was markedly diminished (54.44% relative to YT-NC, normalised to β-actin) in YT cells transfected with miR-223 mimic. YT-NC was adjusted to 100%. Results were quantified by densitometry in 3 independent experiments (mean ± SD) (^#^*P* = 0.008). **(C)** A representative image of PRDM1α protein expression in YT cells as detected by western blot. **(D)** RT-PCR and agarose gel electrophoresis showed ectopic expression of miR-223 with no effect on PRDM1α transcript. NK92, NKL, and K562 cells were transfected with miR-223 inhibitor or NC with HiPerFect Transfection Reagent. **(E)** Compared to NC, the level of endogenous miR-223 was significantly decreased in NK92, NKL, and K562 cells by qRT-PCR analysis. The data are presented as mean ± SE of 4 independent experiments (^∆^*P* = 0.026, ^∆∆^*P* = 0.017, and ^∆∆∆^*P* = 0.044). **(F)** Semi-quantitative analysis by densitometry demonstrated that the PRDM1α protein was restored to 220% and 234% by miR-223 inhibition in NKL and K562 cells, respectively, compared to NC, but the level of PRDM1α protein in NK92 cells was not significantly affected. The data are presented as mean ± SD of 4 independent experiments (^§^*P* = 1.000, ^§§^*P* = 0.040, and ^§§§^*P* = 0.022). **(G)** Representative western blot images of PRDM1α protein levels in NK92, NKL, and K562 cells are shown.

We examined the effect of miR-223 on the PRDM1 protein by transfecting a miR-223 mimic or negative control into YT cells. We used YT cells because they expressed the lowest endogenous level of miR-223 relative to NK92, NKL, and K562 cells. qRT-PCR analysis identified significantly increased level of miR-223 in YT cells transfected with the miR-223 mimic compared to the negative control (Figure 7A, *P* < 0.001). The expression level of the PRDM1α protein decreased to 54.44% in YT cells treated with ectopic miR-223 relative to YT cells treated with the negative control (Figures 7B and C, *P* = 0.008); however, there was no significant difference in the mRNA level of PRDM1α between these 2 groups (Figure 7D), demonstrating that PRDM1α protein expression may be directly downregulated by miR-223 via the inhibition of translation but not by the degradation of PRDM1α mRNA.

### Restoration of PRDM1 expression by reducing miR-223

To test the effect of miR-223 reduction on PRDM1 protein in NK92, NKL, and K562 cells, a miR-223 inhibitor was transfected into cells to reduce the endogenous expression of miR-223. qRT-PCR revealed that the miR-223 inhibitor reduced the levels of endogenous miR-223 in NKL and K562 cells to 40.12% (*P* = 0.017) and 45.10% (*P* = 0.044), respectively, of the negative control (Figure 7E), whereas, the level of PRDM1α protein increased to 220% (*P* = 0.040) and 234% (*P* = 0.022), respectively (Figure 7F). This result provides further experimental evidence that PRDM1α is directly silenced by miR-223. However, we found no distinct changes in PRDM1α expression in NK92 cells (Figure 7F, *P* = 1.000), even though the level of endogenous miR-223 diminished to 55.90% (Figure 7E, *P* = 0.026). Other miRNAs or signals in NK92 cells may regulate PRDM1 expression. Representative images of PRDM1α protein expression in NK92, NKL, and K562 cells are shown in Figure 7G.

### Association of miR-223 with clinical factors of EN-NK/T-NT patients

**Figure 8:**
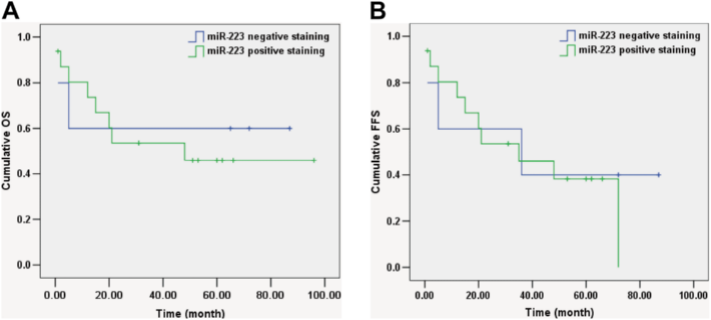
**Kaplan-Meier survival analysis of miR-223 in extranodal NK/T-cell lymphoma, nasal type (EN-NK/T-NT) patients.** According to Kaplan-Meier survival analysis, no correlation was investigated between the expression status of miR-223 and on overall survival (OS) **(A***, P* = 0.784) and failure-free survival (FFS) **(B**, *P* = 0.691) of EN-NK/T-NT patients.

We attempted to analyse the potential biological role of miR-223 expression in 21 EN-NK/T-NT cases. miR-223 positive staining showed no significant correlation with sex, age, tumour stage, or patient status and had no significant effects on the 5-year OS rate, OS, or FFS (Table 2, Figure 8A and B). The lack of a significant association between miR-223 expression and clinical factors in EN-NK/T-NT patients may be due to the limited sample size; future studies should include more patients.

## Discussion

It is becoming clear that PRDM1 functions as a tumour suppressor gene in lymphomas. The inactivation or downregulation of PRDM1 appears to be a common event in activated B cell-like diffuse large B cell lymphoma and is associated with various events including missense mutations, biallelic gene deletions, or the post-transcriptional inhibition of let-7 [19,22,23]. Although research on PRDM1 in NK/T-cell lymphoma is rapidly increasing [18], few studies have examined PRDM1 in Asian EN-NK/T-NT patients, which constitute a large portion of the incidence of this disease in the world.

The present investigation demonstrated that immunostaining of PRDM1 might be prognostic in EN-NK/T-NT. We observed only weak PRDM1 positivity in about one quarter of the EN-NK/T-NT cases (24.59%), consistent with the findings of Iqbal and Karube et al., who reported low levels of PRDM1 expression in NK-cell neoplasms and cell lines compared to normal NK cells [11,13]. However, Ng et al. reported PRDM1 overexpression in 50% (17/34) of NK/T-cell lymphomas [7]. Therefore, studies describing the detection of PRDM1 by IHC are still limited and inconsistent. Because PRDM1 expression could be an important predictor of EN-NK/T-NT, standardisation of immunohistochemical procedures (such as antibodies and the conditions for antigen retrieval and staining evaluation) is necessary to reduce the inconsistency of PRDM1 protein measurements.

In our study, univariate analysis revealed that the frequency of PRDM1-positive expression was significantly higher in early-stage (I/II) than in advanced-stage (III/IV) tumours. We found the advanced stage to be a poor predictor in EN-NK/T-NT cases [2]. Indeed, the important role of PRDM1 in predicting a good outcome is supported by our investigation of its positive effect on patient status, 5-year OS, OS, and FFS in EN-NK/T-NT. The ectopic introduction of PRDM1 in the NK/T lymphoma cell line NKL can induce cell cycle arrest and apoptosis, and the knockdown of PRDM1 in NK cells promotes growth [12,13]. PRDM1 can also promote the apoptosis of tumour cells by specifically suppressing MKI67 and proliferating cell nuclear antigen [24]. In conjunction with previous investigations, our results imply that PRDM1 staining may be used as a positive marker for evaluating the clinical outcome of EN-NK/T-NT patients. However, multivariate analysis demonstrated that PRDM1 expression was not an independent predictor of clinical outcome in our study. This finding may be due to our limited cohort, and we will attempt to enlarge the cohort and perform further analysis of the significance of PRDM1 expression in future studies.

Previous studies primarily attribute the inactivation of PRDM1 to the 6q21 deletion, which occurs in 20 to 43% of EN-NK/T-NT samples and cell lines [3,8,11,12]. Contradicting this view, PRDM1 has been shown to be expressed independent of the presence or absence of the 6q21 deletion [3,11]. In addition, PRDM1 inactivation can be induced by promoter methylation [13]. Ng et al. reported that the expression of PRDM1 can be directly downregulated by miR-30b in NK/T-cell lymphoma [7]. The downregulation of PRDM1 protein in B and T cell lymphomas may be ascribed to different mechanisms. miR-9, let-7a, and miR-30b directly downregulate PRDM1 protein [7,20], and BCL6 and LMP1 repress PRDM1 transcription [25,26]. T-bet and Ets-1 also regulate the expression and function of PRDM1 protein [27,28]. Therefore, based on current knowledge, the inactivation of PRDM1 may be resulted from the 6q21 deletion, DNA methylation, miRNA inhibition, and other distinct signalling pathways. In particular, it has been noted that some cases or cell lines of lymphoma with high levels of PRDM1 mRNA fail to express PRDM1 protein, which implies that post-transcriptional regulation may account for the loss of the PRDM1 protein [3,11,13,19,29]. More importantly, our observations demonstrated the discordance of high PRDM1 mRNA levels and downregulated protein expression in large parts of EN-NK/T-NT cases and some cell lines, increasing the possibility that the steady state of PRDM1 protein may be associated with post-transcriptional regulation.

Our data provide evidence for the downregulation of PRDM1 by miR-223 at the post-transcriptional level as part of the pathogenesis of EN-NK/T-NT. First, the level of the PRDM1 expression was reciprocal to miR-223 expression in EN-NK/T-NT cases or cultured NK/T lymphoma cell lines. Second, miR-223 directly targeted the PRDM1 3′-UTR, as evidenced by luciferase reporter assays. Third, the PRDM1α protein was markedly diminished by the exogenous overexpression of miR-223 in YT cells and restored by miR-223 reduction in NKL and K562 cells, while PRDM1α mRNA was not affected. Thus, the post-transcriptional silencing of PRDM1 by miR-223 might well explain the discrepancy between high PRDM1 mRNA and low protein levels in EN-NK/T-NT found in both our study and in previous reports [3,11,13]; and the targeting of PRDM1 by miR-223 might be an important mechanism of PRDM1 gene inactivation. However, we also noted that the restoration of PRDM1α protein did not occur in NK92 cells; low levels of both PRDM1 transcript and protein were detected in 6 EN-NK/T-NT tissues and NK92 cells, and the methylation in the CpG island of PRDM1 gene reportedly occurs in NK92 cells [11]. Thus, it seems that PRDM1 may be regulated by other parallel regulatory pathways in addition to miR-223.

The identification of miRNAs is a rapidly evolving field, and miRNAs are emerging as central players in the regulation of epigenetic expression [30-32]. The dysregulation of miRNAs has been linked to various types of cancer including lymphocytic malignancy [30,32,33]. miR-223 is located on chromosome Xq12 and plays an essential role in promoting granulocytic differentiation. It is associated with the suppression of erythrocytic differentiation [34-36]. A recent study demonstrated that the overexpression of miR-223 significantly downregulates the mRNA levels of the tumour suppressor gene FBXW7, resulting in an increase in the levels and activity of endogenous cycling E protein and genomic instability [37]. Moreover, higher expression levels of miR-223 correlate with extranodal marginal-zone lymphoma of mucosa-associated lymphoid tissue of the stomach [38]. Markedly increased expression of miR-223 has also been observed in some T-cell acute lymphoblastic leukaemia cases with poor clinical outcomes [39]. Therefore, the function of miR-223 appears to differ in distinct tissues, and these functions may be ascribed to the complexity of the interaction between a miRNA and its target genes and cell type-specific biological effects. Through ISH, we observed specific overexpression of miR-223 in EN-NK/T-NT FFPE samples compared with peripheral T-cell lymphoma and inflammatory nasal mucosa samples. Furthermore, miR-223 directly downregulated expression of the tumour suppressor gene PRDM1, indicating its potential importance in an epigenetic or post-transcriptional role in EN-NK/T-NT.

The mechanism responsible for aberrant overexpression of miR-223 in EN-NK/T-NT is unclear. Although the overexpression of miRNAs in B-cell lymphoma is due to genomic amplification [40], no genomic amplifications or translocations of the Xq12 locus have been reported in several genome-wide analyses of NK/T-cell lymphomas [3,8,11]. The infection of NK cells by EBV is a potential alternative mechanism because nearly all NK/T-cell lymphomas are associated with EBV infection. In our study, all miR-223-positive cases of EN-NK/T-NT showed EBV infection, implying that EBV infection may be responsible for miR-223 overexpression. Indeed, the upregulation of miR-223 has been observed after EBV transformation of lymphoblastoid cells [41]. Motsch et al. [42] also demonstrated that EBV exerts a profound effect on the cellular miRNA profile in EBV-positive NK/T-cell lymphomas compared to non-infected cases. Other reports have revealed that CCAAT/enhancer binding protein alpha and nuclear factor I/A regulate mature miR-223 by competing for a regulatory binding site 700 bp upstream of the pre-miR-223 sequence [43]. Thus, the mechanisms that regulate the level of miR-223 remain to be elucidated.

## Conclusions

Collectively, these findings in our study indicate that PRDM1 is downregulated in EN-NK/T-NT cases and that PRDM1-positive staining may have prognostic value for evaluating the prognosis for EN-NK/T-NT patients. In addition, PRDM1 is likely to be a target of miR-223, and the overexpression of miR-223 might be an important genetic mechanism of PRDM1 downregulation in EN-NK/T-NT. miR-223-mediated silencing of PRDM1 provides new insight into the genetic mechanisms underlying EN-NK/T-NT and an opportunity to identify new therapeutic strategies for EN-NK/T-NT.

## Abbreviations

EN-NK/T-NT: Extranodal NK/T-cell lymphoma, nasal type; miRNAs: microRNAs; PRDM1: Positive regulatory domain containing I; FFPE: Formalin-fixed paraffin-embedded; ISH: In situ hybridisation; EBER-1: Epstein-Barr virus-encoded small RNA; IHC: Immunohistochemistry; qRT-PCR: Quantitative real-time polymerase chain reaction; UTR: Untranslated region; OS: Overall survival; FFS: Failure-free survival.

## Competing interests

The authors have declared that no competing interests exist.

## Authors’ contributions

TL and BZ conceived and designed the experiments. LL, JZ and HT performed the experiments. LL, LN and YD analyzed the data. LN and SZ contributed to reagents/materials/analysis tools. LL, TL, BZ, LN wrote the paper. All authors read and approved the final manuscript.
